# A Literature Review of Microscopic Colitis

**DOI:** 10.7759/cureus.52862

**Published:** 2024-01-24

**Authors:** Ahmed Pervez, Khurram Siddique, Muhammad Amir Saeed Khan

**Affiliations:** 1 General and Colorectal Surgery, Royal Oldham Hospital, Northern Care Alliance NHS Foundation Trust, Oldham, GBR

**Keywords:** management, diagnosis, pathogenesis, clinical features, microscopic colitis

## Abstract

Although the clinical importance of microscopic colitis (MC) is highly increasing, however, the disease is still mysterious due to several challenges. Recent MC data depend mainly on doubts and uncertainties leading to misclassification. This review discussed the current knowledge gaps about MC and various controversies regarding its subtypes, pathogenesis, and management. The diagnosis of MC is based mainly on histology and immunohistopathology which can discriminate two subtypes. However, transitional forms are often associated with misclassification. The site and number of the colon biopsies have been agreed upon as at least three from each side of the colon (right and left) with a total of six. There is no credible, clear explanation for the increased incidence. The etiopathogenesis is possibly multifactorial with a high impact on the immunological background. It is proposed that MC would be the initiative of irritable bowel disease, which needs further data clarification. Although budesonide is an effective treatment in most cases, budesonide-refractory MC represents a significant clinical challenge. Therefore, immunomodulators and biologics are now well-thought to be the second-line choice for treatment.

## Introduction and background

Currently, microscopic colitis (MC) has attracted most gastroenterologists' attention mainly due to its increased incidence as well as its associated knowledge paradox. The clinical importance of MC depends on the apparently intact colonic mucosa during routine endoscopic examination in cases of chronic non-bloody, watery diarrhoea; making it exclusively diagnosed by histopathological examination. Up to 10-20% of chronic diarrhoea is thought to be secondary to MC. The histopathological findings can only distinguish two subtypes of MC. The first subtype is lymphocytic colitis (LC), which is characterized by chronic lymphocyte and plasma cell inflammatory infiltration and proliferation in the lamina propria with epithelium lymphocyte count exceeding 20/100 epithelial cells, while normal values never exceed 5/100 [1].

The second subtype is known as collagenous colitis (CC) in which the subepithelial collagen is thickened more than 10 μm, exceeding the normal thickness of the collagen layer (7 μm). Whenever the histological abnormalities in the two subtypes are only partly detected and do not fulfil the criteria of any subtype despite the clinically suspected MC, the disorder can be called incomplete MC (MCi) [2]. In such a subtype of MC, the histological abnormalities are not indicative because the number of lymphocytes and plasma cells, despite being above the normal upper limit, are still less than 10/100 cells, and subepithelial collagen is also less than 10 μm (below the diagnostic threshold). Therefore, the MCi subtype promotes the scientific doubts that the two main subtypes just represent two distinct different developmental stages of the MC disease. Significantly, MCi patients have the same response to typical treatment for MC as those with complete MC [3].

## Review

Epidemiology

MC is primarily prevalent in females over 60 years old, peaking from the sixth to the eighth decade of life. The CC subtype is nine times more preponderant in females than males, but this sex relationship is lower in the LC subtype [4]. It is important to note that most epidemiological reports about chronic watery diarrhoea caused by MC are mainly collected from high-income countries with a dominant CC subtype. Only a few reports with small sample sizes were collected from low-income and middle-income countries, such as Egypt [5], Peru [6], and Tunisia [7] after colonoscopic biopsy with a predominant LC subtype.

The variances in MC incidence and prevalence in low- and middle-income countries compared with high-income countries mainly referred to the fact that MC diagnosis is dependent only on colonoscopic biopsy and disease awareness among gastroenterologists and pathologists. However, neither the income nor the geography has been reported to impact MC until now. Microscopic CC was first discovered in 1980 with increasing incidence all over the succeeding decades up till now. Such an increase was basically credited to the improved MC awareness and diagnostic biopsies [8].

Risk factors

Diet and Alcohol

Although MC shares the pathogenic mechanisms with coeliac disease, no studies have verified the causal relationship between dietary intake and MC [9]. Previous research work did not correlate alcohol drinking to MC [10,11]; however, a positive relationship between the quantity of alcohol intake and the possibility of MC has recently been reported [9]. Such debate necessitates extra research work to authenticate these potential relations.

Cigarette Smoking

Cigarette smoking has been reported by several studies to be strongly associated with both subtypes of MC with a 2.83-fold increased risk of MC in smokers. The risk of MC was increased with a higher intake of cigarettes and reduced after smoking stoppage [10-14]. Such studies recognized a robust relationship between smoking with CC more than LC. The impact of smoking on MC development may be potentially explained by its effect on stimulating dysbiosis, through up shooting of transforming growth factor-β (TGF-β) which greatly stimulates collagen deposition [15]. Moreover, both humoral and cellular immunity are greatly altered by cigarette smoking leading to damage to the epithelial barrier integrity, hence, contributing to the development of MC [16].

Body Mass Index

Surprisingly, early adulthood obesity has been reported as being protective against MC showing a reverse relationship between the body mass index (BMI) and MC regardless of the resultant weight loss from active MC [17]. The mechanism by which the BMI impacts the development of MC is still indistinct. However, the consequence of low BMI on endogenous sex hormones perhaps elucidates such a link [17].

Gastrointestinal Infections

Enteric infections may participate in the pathogenesis of MC. *Campylobacter *species were accused as a risk factor in the development of MC [18]. However, no study until now had thoroughly assessed specific pathogens as risk of MC warranting additional research to recognize the association between different *Campylobacter *species and MC.

Transplantation of Fecal Microbiota

While using faecal microbiota transplantation (FMT) as a new treatment for resistant *Clostridia difficile *infection, consequent MC cases were reported. This finding denotes that MC may develop following recurrent *C. difficile* infections or microbiome changes secondary to FMT [19].

Diseases association

Notably, MC has been found to be associated with an increased risk of autoimmune disorders, such as coeliac disease, autoimmune thyroiditis, psoriasis, and type 1 diabetes mellitus.

The LC rather than the CC was found to increase the risk of occurrence of coeliac disease by 6.06 times [20]. Therefore, the American Gastroenterological Association and European microscopic guidelines commend screening every case of MC for coeliac disease particularly in cases of resistant diarrhoea in spite of the use of a typical therapeutic regimen of MC [21].

Similarly, MC patients in a national prospective cohort study in Sweden were reported to develop Crohn's disease and ulcerative colitis at a higher rate over time than normal [22]. Fortunately, MC does not appear to be associated with an amplified risk of colorectal cancer as Crohn's disease and ulcerative colitis [23].

However, a slightly increased risk of lymphoma and lung cancer was noted with MC, although this association may be suggested by the robust link between smoking and MC development [24].

Non-steroidal anti-inflammatory drugs

Non-steroidal anti-inflammatory drugs (NSAIDs) are considered critical risk factors for both subtypes of MC. This can be explained by the ability of the NSAIDs to affect the permeability of the epithelial barrier [25] and inhibit the anti-inflammatory prostaglandins regardless of the dose [26].

Proton-pump inhibitors

Several case-control studies have recognized a strong association between the use of high doses of proton-pump inhibitors and the risk of MC. Moreover, the risk of development of CC by proton-pump inhibitors may be stronger than LC as verified by several studies [27-29]. This association can be interpreted by the changes in intestinal pH with proton-pump inhibitors use that alter the tight junction proteins as well as the gut microbiome composition leading to increased epithelial barrier permeability and, hence, the development of MC [29,30].

Selective serotonin reuptake inhibitors

Although several studies have significantly linked between the use of selective serotonin reuptake inhibitors and the risk of MC development, the underlying mechanisms still necessitate extra research. On the other hand, after consideration of other pharmacological agents and lifestyle risk factors, a negative association between selective serotonin reuptake inhibitors and MC has been observed [29].

Mechanisms and pathophysiology

The biological potentials for MC pathogenesis are indistinct and greatly heterogeneous. MC is an inflammatory disorder in the colon of genetically prone persons because of a dysregulated immune response to gut microenvironment alterations [31].

Immune Response

The pathogenesis of CC and LC is characterized by an infiltration of the colonic epithelium by CD8+ lymphocytes and an infiltration of the lamina propria by eosinophils, neutrophils, macrophages, T cytotoxic cells (CD8+) and T helper cells. Such lymphocyte recruitment results in increased expression levels of CXCL9, CXCL10, CXCL11, and CX3CL1 chemokines that are secreted by epithelial cells [32].

Surprisingly, the expression levels of such chemokines persist to be elevated in LC even after remission compared to their diminished levels in CC. Consequently, in active MC subtypes, tissue protein levels of CCL2, CCL3, CCL4, CCL5, CCL7, and CCL22 chemokines are increased to recruit these immune cells and regulatory T cells [33].

This inflammatory infiltration upregulates the pro-inflammatory cytokines involved in the pathways of TH1 and TH17 with marked elevation of IFNy mucosal mRNA levels. The elevated mRNA levels of IFNy, IL-21, and IL-22 are positively associated with the number of everyday bowel activities [34].

Epithelial Barrier Function

Colonoscopic biopsy samples from CC and LC patients have shown a substantial impairment of the transepithelial integrity and increased endocytosis of *Escherichia coli* K12 bacteria through gut-associated lymphoid tissues [35]. The main fundamental mechanism of such impairment is the amplified paracellular permeability, which is mainly controlled by tight junction proteins. Decreased expression of these tight junction proteins (claudin 4, 5, and 8) results in increased paracellular permeability. Remarkably, both mechanistic lanes are strongly controlled by the upregulated IFNy and TNF owing to their role in the internalization of claudin 5 and claudin 8 [36].

Gut Microbiome

Microbial dysbiosis has been greatly supposed to impact the pathogenesis of MC. Such a hypothesis is supported by the histological remission upon faecal stream diversion in refractory MC, followed by recurrence after its reconnection [37]. However, the studies that aimed to characterize specific microbiome alterations in MC are still minor with less consistent data. Reduction of the protective microbiome was accused of exaggerating its activity. Nevertheless, these data need further high-quality research for an improved description of the role of the microbiome in MC pathogenesis [38].

Genetics

Several studies have already reported a robust relationship between the collagenous subtype and human leucocyte antigen (HLA) 8.1 ancestral haplotype. No association was reported between the HLA 8.1 ancestral haplotype and LC subtype despite adequate statistical power, suggesting possible differences in the genetic basis [39].

Bile Acids

Recent research has revealed the presence of abnormal 75-Se-homocholic acid taurine (SeHCAT) scintigraphy, which is a marker of bile acid malabsorption, in both subtypes [40]. This finding supports the implicative role of bile acids in pathogenesis. Besides, a noteworthy reduction in the expression of the main farsenoid X bile acid receptor in the colon of patients was reported to contribute to the pathogenesis through enhancing inflammation and increased paracellular permeability [40].

Collagen Deposition

The main hypothesis in the pathology of the collagenous subtype is the imbalance between the subepithelial type III and VI collagen, with small amounts of type I collagen production and breakdown. Therefore, TGF-β, a powerful collagen deposition stimulator, showed increased expression [37,38]. On the other hand, the upregulation of TGF-β with subsequent collagen deposition forming fibrotic reaction following inflammatory response or microbiome changes is also suggested. Moreover, endoscopic samples of patients with CC showed greater expression of TIMP metallopeptidase inhibitor 1 (TIMP1) from myofibroblasts, which greatly impairs the extracellular matrix lysis [40].

Clinical manifestations and natural history

The major clinical complaint is chronic, watery, non-bloody diarrhoea that might be accompanied by painful abdominal cramps. Remarkably, in contrast to Crohn's disease and ulcerative colitis, bloody diarrhoea does not occur. The classical course usually involves an alternation between normal bowel habits to severe diarrhoea. Weight loss, dehydration, faecal incontinence and urgent nocturnal diarrhoea can occur commonly in MC [40].

The symptoms may reoccur in 80% of patients within three months after the stoppage of traditional treatment. Diarrhoea may be recurrent years after spontaneous remission but without showing an increased mortality or colon cancer morbidity. Therefore, it can be described as relapsing and remitting disease as some patients may relapse after developing steroid tolerance. Hjortswang criteria were developed to define clinical remission in patients with already diagnosed MC, in the form of a mean of less than three stools and less than one watery stool per day over a given week [36,40].

Clinical scoring systems were developed to overcome the overlap between irritable bowel syndrome and associated chronic diarrhoea to facilitate the pre-colonoscopy probability. The clinical scores predicting the risk take into consideration the lifestyle factors, old age ≥55 years, chronic diarrhoea not less than six months, bowel movements more than five per day, low BMI <30 kg/m^2^, smoking, and pharmacological use of NSAIDs or selective serotonin reuptake inhibitors [30,31,37].

Notably, assessing the disease severity was also developed by indexing the most reported symptoms regarding the number of unformed daily diarrhoea, nocturnal diarrhoea, abdominal pain, weight loss, stool urgency and incontinence [37]. Till now, there are no reported complications except for recurrence. However, long-term treatment with budesonide would be associated with multiple complications [38].

Diagnosis

The diagnosis is mainly found and confirmed by histological examination after colonoscopic biopsy as the clinical symptoms overlap with many conditions that should be considered in the differential diagnosis, especially irritable bowel syndrome. Unfortunately, neither laboratory abnormalities nor faecal biomarkers are diagnostic. Therefore, pre-colonoscopic biopsy should be preceded by thorough history taking considering probable risk factors. The global differences in diagnosis between developing and developed countries are mainly dependent on disease awareness between gastroenterologists, endoscopists, and pathologists [40].

Laboratory testing

Till now, no laboratory stool tests have been recommended for diagnosis despite being numerous. Faecal calprotectin levels in patients with active disease have been shown to be greatly elevated in comparison with its low levels in patients with irritable bowel syndrome. Yet, the faecal calprotectin level is still lower than its levels in other inflammatory colon disorders making its use as a diagnostic technique misleading [34]. Several other faecal biomarkers have been reported to be elevated including lactoferrin, eosinophil protein X, eosinophil cationic protein, and enteroendocrine markers (chromogranin A, chromogranin B, and secretoneurin) [40]. Nearly half of the cases show a high erythrocyte sedimentation rate, mild anaemia as well as positive autoantibodies to rheumatoid factor, antinuclear antibodies, antimitochondrial antibodies, and antithyroid antibodies [40]. Further diagnostic studies for the evaluation of MC cases should exclude the presence of *C. difficile* toxin, *E. coli* 0157: H7, protozoal stages, and transglutaminase IgA antibody for celiac disease [32].

Endoscopic findings

Although the colon appears endoscopically normal or nearly normal, high-definition endoscopy can reveal oedema, patchy erythema, nodularity, petechiae, and rarely ulceration. Notably, the effect of NSAIDs should be considered in histologically confirmed patients with ulcerations. With the presence of pseudomembranes, ulcers and collagen thickening in the affected colon, appropriate selection of the site of the biopsies is crucial to avoid any diagnostic doubt. Therefore, at least six to eight biopsies from random colon segments both right and left colon are essential for accurate diagnosis [33].

Histological criteria

The two subtypes share remarkable histopathological similarities. The epithelium in the lymphocytic subtype is characterized by mild surface damage with increased lymphocytes, plasma cells, eosinophils, and neutrophils in the lamina propria with little or no crypt alterations on haematoxylin and eosin staining. In the collagenous subtype the eosinophils, neutrophils, and crypt abscesses are the most observed histological hallmarks. In patients with incomplete disease, CD3 immunohistochemical staining of the lymphocytes can be used for differentiation in most cases [34]. Immunohistochemistry and connective tissue staining are highly recommended in borderline cases and in patients with difficult diagnoses. In both subtypes, the lamina propria inflammation is more noticeable in the proximal part of the colon than the distal one. Likewise, the subepithelial collagen band appears thicker in the proximal colon than in the distal one in the collagenous subtype. Based on such data, a full ileocolonoscopy (ascending, transverse, descending, and sigmoid) with at least two biopsies per section is crucial for accurate diagnosis [35].

Screening and prevention

Although the major lifestyle risk factors and pharmacological associations are well known, proactive screening and protective measures show no clear value and are not currently recommended. However, all confirmed cases should be investigated for coeliac disease [40].

Management

Presently, the FDA is not approving any treatment; however, the European Medicines Agency approved only oral budesonide therapy. Clinical trials defined the clinical response to treatment as a decrease in stool rate by 50% or more, clinical improvement, less than three stools per day, and less than one watery stool per day. Similarly, histological improvement is defined as a decrease of intraepithelial lymphocytes of less than 20/100 epithelial cells in the lymphocytic subtype and a collagen band thickness of 10 μm or less in the collagenous subtype with the absence of lamina propria inflammation. Patients with MC who have been clinically improved are not in need of histological remission confirmation by endoscopy [36].

Medication Withdrawal and Lifestyle Changes

A few studies have accused smoking and several medications of causation; however, treatment procedures including quitting smoking and stoppage of such medications have been reported to be of low value. Very few reports have revealed remission after lansoprazole, ticlopidine, and NSAIDs cessation. Additional studies are necessary to define the role of medication.

Antidiarrheal Medications

Loperamide was reported to have a 71% efficacy rate, raising the consensus that antidiarrheals can be used alone in mild disease or in combination with other treatments in moderate to severe MC to reduce the occurrence of diarrhoea [38].

Budesonide

According to high-quality data, budesonide is considered the gold standard treatment in a dose of 9 mg/day. Budesonide is a second-generation corticosteroid with extensive first-pass metabolism in the liver, which can minimize systemic glucocorticoid activity and subsequent side effects. Budesonide-induced adverse effects are generally rare including headache, back pain or arthralgias, weight gain, hypertension, hyperhidrosis, and psychiatric effects. Fortunately, its long-term use was not associated with an increased incidence of osteoporosis [31].

Recent, high-quality evidence indicates that more than 80% of cases are clinically and histologically improved within six to eight weeks of budesonide treatment. Budesonide can bind locally with a high affinity to intracellular glucocorticoid receptors at the site of inflammation 195 and 15 times higher than that of hydrocortisone and prednisolone, respectively [32].

One of the most common presentation forms of budesonide is available in delayed-release capsules containing lactose and coated by a protective ethyl-cellulose matrix that dissolves at pH > 6.4. Such enteric formula permits selective absorption of budesonide from the distal ileum and colon [33].

Notably, low-dose budesonide maintenance treatment of 6 mg daily is essential to avoid relapse occurring largely in 60-80% of CC cases after drug termination. Budesonide is more effective than other anti-inflammatory and anti-secretory drugs including prednisolone, mesalazine, cholestyramine, bismuth subsalicylate, and loperamide [33,34].

Other Corticosteroids

Recently, an extended-release, multi-matrix formulation of budesonide (MMX-B) has been developed for refractory disease to the standard budesonide treatment. It can deliver budesonide all over the colon with promising results in most cases [34]. Beclomethasone dipropionate is another colonic, locally released steroid that has also been studied in refractory disease showing remarkable clinical improvement after eight weeks of therapy [35]. In addition to the locally released steroids, systemic prednisone or prednisolone have also been investigated for treatment for two weeks with restricted clinical remission than obtained from budesonide treatment in all cases [35].

Mesalamine

Mesalamine was one of the most used therapies in patients. However, its use in the collagenous subtype is not recommended in state-of-the-art strategies owing to its lower efficiency compared to budesonide as regards both clinical and histological remission. Also, mesalamine use has been associated with many side effects, mainly pancreatitis, elevated hepatic enzymes, metatarsalgia, and dizziness [36,40].

Bile Salt-Binding Agents

As bile acids are implicated in pathogenesis, bile salt-binding agents are recommended to help in its management. Nevertheless, only low-quality data about their success is reported. Cholestyramine or colestipol therapy led to remission within one week in most patients, especially in cases with bile acid malabsorption. Bile salt-binding agents can be used for the management of budesonide-refractory disease and as adjunctive agents with mesalamine to induce faster remission in both subtypes [37,38].

Refractory Disease

Although being the first-line therapy in most patients, the budesonide treatment course is complicated by relapse in more than 80% of patients and ineffectiveness in about 7% of cases. Numerous regimens have been recently developed to treat budesonide-refractory and budesonide-dependent patients. Immunomodulators including azathioprine, mercaptopurine, and methotrexate are still considered the standard of care in such cases up till now but with low-quality of evidence [29-32]. Adverse drug events may occur in patients treated with these agents. Despite symptomatic control of patients naive to budesonide, weekly subcutaneous methotrexate was associated with severe side effects discouraging its use in MC management [32].

Recently, infliximab, a tumour necrosis factor-a antagonist, has been recommended for budesonide-refractory and budesonide-dependent cases because of its ability to induce clinical remission after the first dose in both MC subtypes [33,34]. Vedolizumab and adalimumab have been shown to produce fewer side effects relative to infliximab and maintain remission for more than one year [35-37].

Probiotics

A few studies investigated faecal microbiota transfer as well as some probiotics (e.g., *Boswellia serrata*, *Lactobacillus acidophilus* LA-5, and *Bifidobacterium animalis* AB-Cap-10 in the management of MC. Most studies failed to show significant benefit or achieved only partial clinical and histological remission [37]. Still, large, randomized, controlled trials are mandatory to accurately specify the efficacy, if any, of these therapeutic lines in the management of MC.

Surgical Ileostomy

Faecal diversion with a diverting ileostomy had promising findings for the control of MC in medication-refractory patients. Such a surgical procedure is considered the last choice in refractory cases not responding to medication therapy. The success of such a technique in inducing histological remission highlights the implicative role of the microbiome or faecal stream in the pathogenesis of the disease [32]. The diversion of the faecal stream improved the epithelial barrier dysfunction and permeability and normalized the elevated cytokines levels. As reversing the technique is typically accompanied by disease relapse, this tactic is advised to be permanent ileostomy [34].

Quality of life

Several studies have reported the negative effect on the patients' health-related quality of life especially comorbid fatigue, anxiety, and depression due to bile acid malabsorption. As symptoms overlap with inflammatory bowel disease (IBD) and irritable bowel syndrome (IBS), the condition remains underdiagnosed and patients suffer unfortunately. This is also attributed to a lack of awareness among health professionals and a lack of an accurate diagnostic test. Fecal calprotectin which happens to be the cornerstone for diagnosis of IBD and IBS cannot diagnose MC. Symptoms include urgency, frequency, watery diarrhoea, and incontinence which is shown with high St. Mark's Incontinence Scores. These effects may even remain after remission [35]. However, successful therapy of MC has a direct impact on the patients' social function, disease-related worry, and general well-being, especially after treatment with budesonide.

## Conclusions

MC is characterized by chronic, intermittent diarrhoea which can sometimes resolve without treatment or within weeks of treatment. Relapses are very common but with no increased risk of colorectal malignancy. Despite increasing awareness and knowledge, various queries remain unanswered due to contradictory data. These queries regarding many features as well as management of the disease suggest further investigation.
